# U. S. V. M. A.

**Published:** 1897-06

**Authors:** 


### U. S. V. M. A.

NASHVILLE, TENN., SEPTEMBER 7, 8, AND 9, 1897.

Secretary Stewart has sent out a timely letter to those of the members who have become delinquent in dues, and we are sure it will bring many responses and create fresh interest in the coming meeting at Nashville.

Among those who will present papers at Nashville we learn with pleasure that Dr. E. A. A. Grange, of Michigan, will offer one on "Infectious Mammitis."

The programme at Nashville will announce papers by Drs. W. L. Williams, M. H. Reynolds, E. P. Niles, and S. J. J. Harger.

Dr. A. S. Wheeler, formerly of New Orleans, now at Biltmore Farm, N. C. (the change necessary on account of ill health), writes me in response for a paper, that if he finds that he can attend he may prepare a paper for the Nashville programme.

The "Bureau of Animal Industry's" display consists of models and specimens in alcohol representing some of the infectious diseases in the domesticated animals, models of diseased horses' hoofs, shoes for the correction of faulty gaits, and the treatment of diseases of the hoof; tags, implements, etc., used in the United States meat-inspection service; cultures of bacteria, toxins and antitoxins, animal parasites; a pyramid of wood samples from the Southern States; poultry illustrating the gape-worm disease; photographs of famous horses and cows. The exhibit was prepared by Dr. D. E. Salmon, Chief of the Bureau, assisted by Dr. C. F. Dawson.

Dr. W. H. Dalrymple, of Louisiana, has consented to read a practical paper on a popular topic at the Nashville meeting.

